# Reliability and sensitivity of mechanical properties of phase-specific during the 505-test using a radar device in soccer players

**DOI:** 10.3389/fphys.2026.1840593

**Published:** 2026-05-29

**Authors:** Jin Li, Hongxing Li, Fan Gu, Ying Li, Ruiqi Cheng, Lin Song, Christophe Hautier, Qingshan Zhang

**Affiliations:** 1 Department of Physical Education, Fujian Business University, Fuzhou, China; 2 College of General Education, Fujian Preschool Education College, Fuzhou, China; 3 Department of Public Physical Education, Minjiang University, Fuzhou, China; 4 School of Athletic Performance, Shanghai University of Sport, Shanghai, China; 5 Universite Claude Bernard Lyon 1, LIBM, Inter-University Laboratory of Human Movement Biology, UR 7424, Villeurbanne, France

**Keywords:** COD, radar device, reliability, mechanical properties, young soccer

## Abstract

Change-of-direction (COD) ability is a key determinant of soccer performance. This study evaluated the intra- and inter-day reliability and sensitivity of a single-radar device (SRD) for capturing phase-specific kinetics and kinematics during the 505 tests in 24 youth soccer players. Intra-day results showed high reliability for maximum velocity (Vmax, ICC = 0.67, CV = 1.51%) and moderate-to-high reliability for kinetic variables (P2_HBF avg and P3_HAP avg, ICC = 0.58–0.59). However, all intra-day metrics exhibited “Marginal” usefulness as Typical Error (TE) consistently exceeded the Smallest Worthwhile Change (SWC). In contrast, inter-day reliability was substantially higher, particularly for kinetic indicators such as braking impulse (P2_HBI avg, ICC = 0.90) and re-acceleration force (P3_HAF max, ICC = 0.91). Furthermore, inter-day assessment revealed “Good” usefulness (TE≤SWC) for key metrics, including P2_HBIavg, P3_HAFavg/max, and re-acceleration distance/velocity. These results indicate that while intra-day stability is limited by movement variability, RRDs provide sufficient precision for individualized monitoring across testing days. Practitioners should prioritize inter-day kinetic profiles and incorporate familiarization to maximize diagnostic accuracy in youth soccer populations.

## Introduction

The ability to execute a rapid change of direction (COD) is an essential physical capacity required in many multidirectional sports (Dos’Santos et al., 2020; Zhang et al., 2022). Previous studies have indicated that COD ability is a crucial determinant of performance in soccer, influencing players’ capacity to execute rapid maneuvers under dynamic match conditions (Nimphius et al., 2018; Harper et al., 2022). The superior ability to perform complex COD movements, such as cutting, pivoting, and turning, is frequently required during competitive play and is crucial to determining the success of offensive and defensive actions, thereby enabling players to surpass opponents in decisive moments (Jm and Wb, 2006). Accurate assessment and enhancement of COD ability are therefore central to modern talent development and injury prevention strategies.

To understand the mechanics of this capacity, COD can be defined as a multidirectional action that includes rapid accelerations, intense decelerations, the moment of directional shift, and subsequent re-acceleration (Hewit et al., 2013; Harper et al., 2022). Thus, effective COD performance is intrinsically linked to an athlete’s ability to accelerate and decelerate efficiently (Hewit et al., 2013; Zhang et al., 2022). Emerging research has emphasized that deceleration phases are particularly biomechanically demanding, placing substantial neuromuscular stress on the lower limbs (Harper et al., 2022; Zhang et al., 2022; Philipp et al., 2024). Notably, deceleration demands in soccer can exceed those of acceleration, with play direction (Spiteri et al., 2015; Philipp et al., 2023). Given these complexities, understanding the kinetic and kinematic parameters underlying these movement phases is vital for optimizing COD performance and mitigating injury risk (Harper et al., 2024). Furthermore, the ability to decelerate effectively is often a limiting factor in COD efficiency, which directly impacts performance in agility-based sports such as soccer (Harper et al., 2022; Zhang et al., 2022).

The biomechanical complexity of COD maneuvers has driven a methodological shift from traditional timing gates toward technology capable of capturing continuous mechanical variables (e.g., velocity, force, and power) (Harper et al., 2021, 2022; Zhang et al., 2022). Recent technological advancements have enabled researchers to obtain more detailed assessments of athletes’ phase-specific COD ability across laboratory and field-based settings. For instance, motorized resistance devices (MRD)have been applied to COD testing to provide continuous velocity measurement, offering phase-specific information while prescribing horizontal load (Rakovic et al., 2022). However, motorized resistance technology requires attaching a cable to the body, which may alter natural movement kinematics, affect mobility, and limit its practical feasibility during high-speed maneuvers.

As a more ecologically valid alternative, single radar devices (SRD) provide a non-invasive, real-time assessment of kinematic profiles, such as velocity-time curves, making them particularly suitable for field-based evaluations (Perez et al., 2021; Harper et al., 2022; Zhang et al., 2022). Additionally, the precise measurements of kinetic (force-related) and kinematic (movement-related) variables can be computed in real time within field-based environments using the general mechanic’s model. Previous studies have shown the high reliability of measuring mechanical properties—such as force, velocity, power, acceleration, and deceleration—computed from the velocity-time curve using a general mechanical model during linear acceleration and deceleration maneuvers (Zhang et al., 2022; Harper et al., 2023). For instance, while recent work by Harper et al. (2023) found that the radar device showed higher reliability in measuring maximal braking performance and mechanical properties of linear deceleration (Harper et al., 2023), the biomechanical complexity of a 180° turn poses unique signal-tracking challenges (Zhang et al., 2022; Harper et al., 2023). Also, the reliability of radar-derived measurements during multidirectional, COD-specific tasks remains underexplored.

The 505-test, widely recognized for its simplicity and relevance to soccer-specific COD demands, provides an ideal framework for validating radar-based assessments (Rakovic et al., 2022). Since no study has comprehensively examined the reliability of radar devices in capturing discrete phase-specific mechanical properties during 180-degree turn COD assessments, establishing this validation is crucial for practitioners to use radar technology for performance assessment and training interventions with confidence. The differentiation between kinematic stability and kinetic stability during such complex turns remains a critical methodological question. Moreover, a notable gap remains in evaluating phase-specific mechanical properties during COD tasks, especially within youth soccer populations that exhibit unique neuromuscular characteristics.

Therefore, this study aims to investigate the intra-day and inter-day reliability of a radar device in measuring phase-specific mechanical properties during the 505 COD test in younger soccer players. By quantifying the stability of both kinetic and kinematic outputs, this research seeks to establish the practical utility of radar technology in youth athletic diagnostics. It was hypothesized that i) the kinetic parameters (e.g., force, power, impulse) during the COD test would exhibit higher reliability due to their integrated nature, while ii) the kinematic parameters (e.g., time and distance) might indicate lower reliability owing to the inherent movement variability of youth athletes.

## Materials and methods

### Participants

Twenty-four male youth soccer players (age: 14.6 ± 0.8 years; height: 1.68 ± 0.06 m; body mass: 58.2 ± 5.3 kg), competing at a regional competitive level, voluntarily participated in this study. All participants had at least 7 years of structured soccer training experience. Players were free of musculoskeletal injuries in the past 6 months and provided written informed consent, with parental approval. This study was approved by the local Institutional Ethics Committee (Reference: SYLL20231101). All procedures were conducted in accordance with the principles outlined in the Declaration of Helsinki. Written informed consent was obtained from all participants after they received a comprehensive verbal and written explanation of the study’s purpose, procedures, and potential risks.

### Study design

A cross-sectional study design was employed to assess the reliability and sensitivity of a radar device in measuring phase-specific mechanical properties (acceleration, deceleration, re-acceleration) during the 505-COD test. Participants performed two testing sessions, separated by 72 hours, under identical environmental conditions to assess test-retest reliability. Additionally, timing gate measurements were used concurrently as a reference standard to validate temporal accuracy (e.g., split time) assessment.

### Testing procedure

Before testing, all participants performed a standardized warm-up consisting of 5 minutes of jogging, dynamic stretching, and submaximal COD trials. However, it is important to note that no separate, dedicated familiarization session was conducted on a preceding day. Following the warm-up, participants completed three valid trials of the 505-COD test on their right turning leg, with a 2-minute passive recovery period between trials. The 505-COD test protocol was conducted in accordance with established guidelines (Zhang et al., 2022). The participant was instructed to start from a two-point stance positioned 0.5 m behind the line, sprint maximally to the turning point, execute a 180° turn by planting the right foot, and then reaccelerate for a 5 m sprint to the finish. A Doppler radar gun (Stalker ATS II, Applied Concepts Inc., USA) was utilized to capture instantaneous velocity data at a sampling frequency of 46.9 Hz; while standard for this field-based device, this frequency may limit the resolution of rapid kinetic transient peaks during the complex change of direction movement. The radar gun was mounted on a tripod and positioned to ensure direct alignment with the participants’ sagittal plane during the test. The radar device was placed 5 meters behind the starting line, at a height of ~0.9 meters, and was aligned horizontally with the athlete’s center of mass. A dual-beam electronic timing gates system (Witty, Microgate^®^, 1000 Hz, Witty, Bolzano, Italy) was used as the criterion measure for COD performance times. Gates were placed at the start line and 10 m finish line to capture split times. After ~6 minutes of recovery, participants performed 505 tests from a crouched position (staggered stance), with a ~4-minute recovery period per trial. Trials were deemed valid when players planted their foot beyond the designated turning line with complete foot contact.

### Data processing and variable calculation

All data were collected using the software Stalker Acceleration Testing System provided by the radar device’s manufacturer. Following procedures outlined by Harper (Harper et al., 2023), the processing involved: (i) trimming data to include only the relevant test duration, (ii) ensuring the velocity-time curve commenced from zero, and (iii) applying a digital fourth-order, Butterworth filter with a cut-off frequency of 1.5 Hz to the raw velocity data to minimize high-frequency noise. Instantaneous acceleration was derived from the filtered velocity data using a finite difference method (Central Difference). Custom data analysis programs (MATLAB, R2023b, Natick, MA, United States) were used to compute kinematic and dynamic variables.

**Figure 1:**
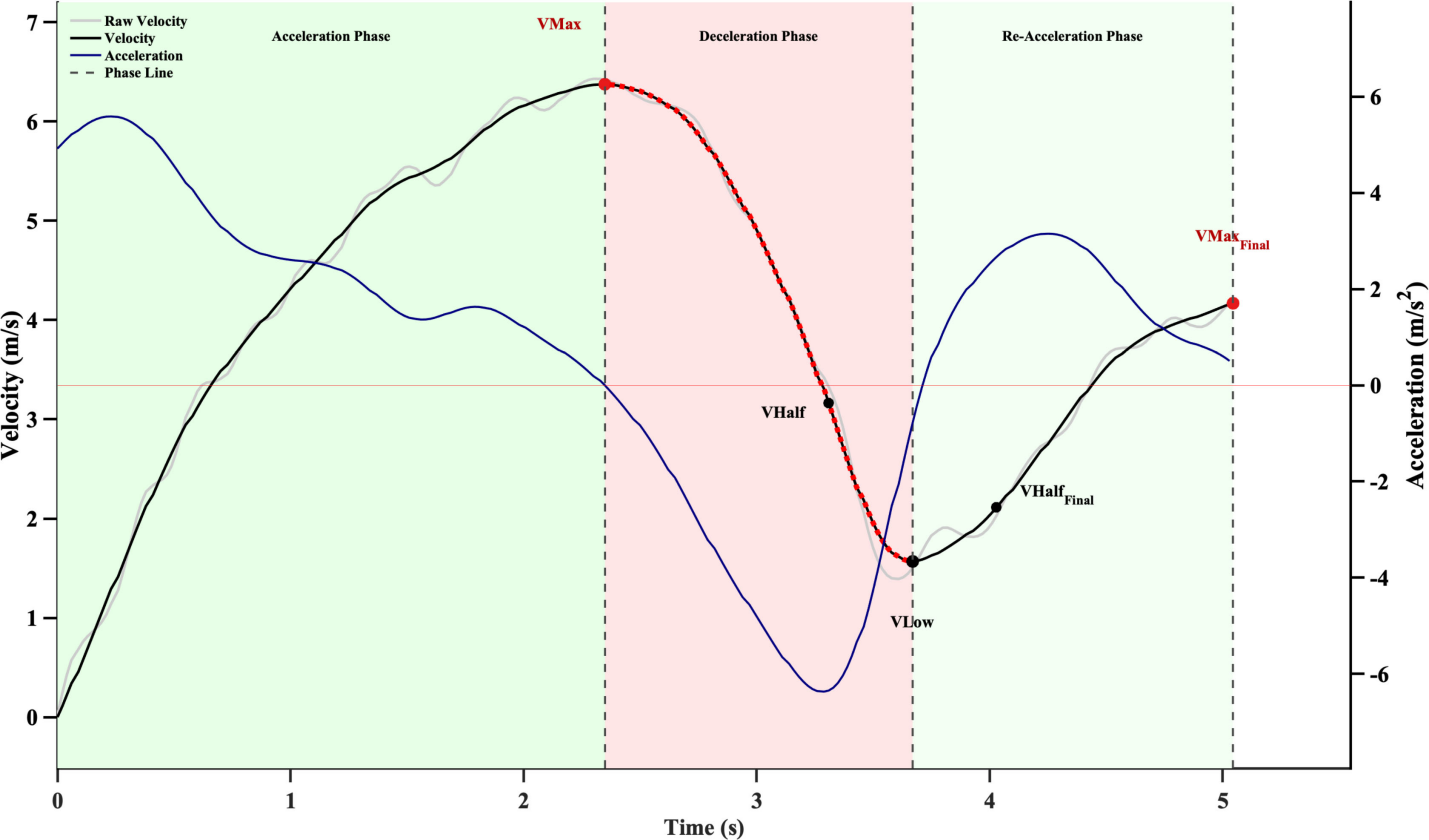
Typical velocity-time profile of the 505-test illustrating the definition of initial acceleration, deceleration, and re-acceleration phases.

Instantaneous acceleration was calculated from the filtered velocity data using a five-point difference method, and the acceleration data were upper- and lower-clipped (± 12 m/s²) to eliminate outliers. Based on the characteristics of the velocity-time curve, the 505 test is divided into four key phases from a kinematic perspective (**Figure 1**), with specific definitions provided in **Table 1**.

**Table 1:** Definitions and abbreviations of the 505 change-of-direction test phases.

Phases	Subphases level	Abbreviation	Definition or calculation
Phase 1	Initial acceleration	In_Acc_ Phase	Initial acceleration is defined as the maximum velocity (Vmax) reached during the first phase from the start of measurement.
Phase 2	Deceleration	Dec_Phase	Deceleration phase is defined as the period from Vmax to Vlow
Phase 3	Re-acceleration	Re_Acc_Phase	Re-acceleration phase is defined as the period from Vlow to Vmax_finish

The kinetic variables are used in a previously estimated general inverse dynamic model that incorporates Newton’s second law and accounts for air resistance. The formula for calculating the instantaneous horizontal net force $F_{net}$ is as follows:


$$F_{net}(t)=m\cdot at+F_{air}(t)$$

where *m* is body mass and a(t) is instantaneous acceleration. Air resistance $F_{air}\;$was estimated as:


$$F_{air}(t)=0.5\cdot \rho \cdot A_{f}\cdot C_{d}\cdot v{\left(t\right)}^{2}$$

with air density $\rho =1.225kg/m^{3}$ and drag coefficient $C_{d}=0.9$.The participant’s frontal area ( $A_{f}$) was estimated based on height and mass using the Du Bois body surface area formula

Instantaneous horizontal power ( P) was calculated as the product of force and velocity:


$$P(t)=F_{net}(t)\cdot v(t)$$

Horizontal impulse ( J) was calculated by integrating the instantaneous force with respect to time (trapezoidal integration):


$$J=\int_{t_{start}}^{t_{end}}F_{net}(t)\cdot dt$$

Kinetic variables were categorized based on the phase of movement (**Figure 1**, **Table 1**): The kinematic variables during the Acceleration phase include Time, Distance, Average velocity, Maximum velocity, Time to maximum velocity, Average acceleration, and Maximum acceleration. The kinetic variables during the Re-acceleration phase include Average Horizontal Acceleration Force ( $HAF_{avg}$), Maximum Horizontal Acceleration Force ( $HAF_{max}$), Average Horizontal Acceleration Impulse ( $HAI_{avg}$), Average Horizontal Acceleration Power $\left(HAP_{avg}\right)$, and Maximum Horizontal Acceleration Power $\left(HAP_{max}\right)$ (**Table 2**). The kinematic variables during the deceleration phase include Time, Distance, Average deceleration, Maximum deceleration, Time to maximum deceleration, and Distance to maximum deceleration. The kinetic variables during the deceleration phase include: Average Horizontal Braking Force ( $HBF_{avg}$), Maximum Horizontal Braking Force ( $HBF_{max}$), Average Horizontal Braking Impulse ( $HBI_{avg}$), Average Braking Power ( $HBP_{avg}$), and Maximum Braking Power ( $HBP_{max}$) (**Table 2**).

**Table 2:** Descriptions and calculation methods of phase-specific kinematic and kinetic outcome variables.

Phase	Variable category	Variable	Abbreviation	Definitions or calculations
Phase 1 (Initial acceleration)	Kinematic	Time (s)	P1_Time	Time in Initial acceleration phase
		Distance (m)	P1_Dist	Distance in Initial acceleration phase
		Average velocity(m·s^-1^)	P1_Vavg	Average of all instantaneous velocity values during the Initial acceleration phase
		Maximum velocity (m·s^-1^)	P1_Vmax	Maximum instantaneous velocity measurement during the Initial acceleration phase.
		Average acceleration (m·s^-^²)	P1_Accavg	Average of instantaneous acceleration values during the Initia acceleration phase.
		Maximum acceleration (m·s^-^²)	P1_Accmax	Maximum instantaneous acceleration measurement during the Initia acceleration phase
Phase 2 (Deceleration)	Kinematic	Time (s)	Dec_Phase_Time	Time in deceleration phase.
		Distance (m)	Dec_Phase_Dist	Distance covered in the deceleration phase.
		Average deceleration (m·s^-^²)	Dec_Phase_Decavg	Average of all instantaneous deceleration values during the deceleration phase
		Maximum deceleration (m·s^-^²)	Dec_Phase_Decmax	Maximum instantaneous deceleration during the deceleration phase
		Time to maximum deceleration (s)	Dec_Phase_Decmax_Time	Time to maximum deceleration from the start of the deceleration phase.
		Distance to maximum deceleration (m)	Dec_Phase_Decmax_Dist	Distance to maximum deceleration from the start of phase 1a.
	Kinetic	Average horizontal braking force (N)	Dec_Phase_HBFavg	Average of all instantaneous horizontal braking force (HBF) values during the deceleration phase.
		Maximum horizontal braking force (N)	Dec_Phase_HBFmax	Maximum instantaneous HBF value during the deceleration phase
		Average horizontal braking impulse (N·s)	Dec_Phase_HBIavg	Average horizontal braking impulse (HBI) calculated from the instantaneous momentums (F • t=m • Δv) during the deceleration phase.
		Average braking power (W)	Dec_Phase_HBPavg	Average of all instantaneous horizontal braking power (HBP) values obtained during the deceleration phase
		Maximum braking power (W)	Dec_Phase_HBPmax	Maximum instantaneous HBP value during the deceleration phase.
Phase 3 (Re-acceleration)	Kinematic	Time (s)	ReAcc_Phase_Time	Time in re–acceleration phase.
		Distance (m)	ReAcc_Phase_Dist	Distance covered in the re–acceleration phase.
		Average velocity(m·s^-1^)	ReAcc_Phase_Vavg	Average of all instantaneous velocity values during the re–acceleration phase.
		Maximum velocity (m·s^-1^)	ReAcc_Phase_Vmax	Maximum instantaneous velocity measurement during the re–acceleration phase.
		Average acceleration (m·s^-^²)	ReAcc_Phase_Accavg	Average of instantaneous acceleration values during the re–acceleration phase.
		Maximum acceleration (m·s^-^²)	ReAcc_Phase_Accmax	Maximum instantaneous acceleration measurement during the re–acceleration phase.
	Kinetic	Average horizontal acceleration force (N)	ReAcc_Phase_HAFavg	Average of all instantaneous horizontal acceleration force (HAF) values during the re–acceleration phase.
		Maximum horizontal acceleration force (N)	ReAcc_Phase_HAFmax	Maximum instantaneous HAF during the re–acceleration phase.
		Average horizontal acceleration impulse (N·s)	ReAcc_Phase_HAIavg	Average HAI calculated from the instantaneous momentums (F • t=m • Δv) during the re–acceleration phase.
		Average horizontal acceleration power (W)	ReAcc_Phase_HAPavg	Average of instantaneous horizontal acceleration power (HAP) values during the re–acceleration phase.
		Maximum horizontal acceleration power (W)	ReAcc_Phase_HAPmax	Maximum instantaneous HAP value during the re–acceleration phase.

### Statistical analysis

Data normality was assessed using the Shapiro–Wilk test. Descriptive statistics are presented as mean ± standard deviation (SD) with 95% confidence intervals (CI). Intra-day and inter-day reliability were quantified using the intraclass correlation coefficient (ICC, two-way random, absolute agreement) and the coefficient of variation (CV%). ICC values were interpreted as: low (<0.50), moderate (0.50–0.75), high (0.75–0.90), and nearly perfect (>0.90) (Hopkins, 2000; Koo and Li, 2016). Measurement error was further evaluated using the standard error of measurement (SEM) and the typical error (TE). To assess the usefulness (sensitivity) of each metric, the TE was compared with the smallest worthwhile change (SWC), calculated as 0.2 times the between-subject SD. The usefulness was categorized as: “Good” (TE ≤ SWC), “Satisfactory” (TE slightly > SWC), or “Marginal” (TE > SWC) (Hopkins, 2000; Koo and Li, 2016). Concurrent validity was assessed by calculating Pearson’s correlation coefficients (r) between radar-derived metrics and timing gate measures. Bland–Altman plots were constructed to assess agreement and systematic bias. All statistical procedures were performed in R (version 4.2.0 or later), with a significance level of p < 0.05.

## Results

The intra- and inter-day reliability and sensitivity results are summarized in **Tables 3**, **4**.

**Table 3:** Intra-day reliability and sensitivity of phase-specific kinematic and kinetic metrics during the 505 change-of-direction test.

Variable	Trial 1	Trial 2	ICC (95% CI)	ICC magnitude	CV%	SEM	TE	SWC	Usefulness
P1_Time	2.46 ± 0.24	2.39 ± 0.14	0.23 (0.01,0.57)	Low	9.82	0.15	0.24	0.08	Marginal
P1_Dist	3.05 ± 0.25	3.09 ± 0.22	0.15 (0.07,0.5)	Low	7.40	0.13	0.23	0.07	Marginal
P1_Vavg	4.08 ± 0.25	4.23 ± 0.2	0.36 (0.1,0.67)	Low	4.67	0.13	0.20	0.08	Marginal
P1_Vmax	6.28 ± 0.16	6.2 ± 0.15	0.67 (0.43,0.87)	Moderate	1.51	0.08	0.09	0.07	Marginal
P1_max_Time	2.46 ± 0.24	2.39 ± 0.14	0.23 (0.01,0.57)	Low	9.82	0.15	0.24	0.08	Marginal
P1_Accavg	2.57 ± 0.27	2.61 ± 0.18	0.37 (0.1,0.68)	Low	7.97	0.14	0.21	0.09	Marginal
P1_Accmax	5.21 ± 0.98	6.18 ± 1.65	0.44 (0.18,0.73)	Low	19.02	0.82	1.13	0.55	Marginal
P2_Time	1.34 ± 0.21	1.32 ± 0.12	0.34 (0.08,0.67)	Low	9.06	0.09	0.12	0.05	Marginal
P2_Dist	1.79 ± 0.33	1.72 ± 0.17	0.25 (0.01,0.58)	Low	11.28	0.12	0.19	0.07	Marginal
P2_Decavg	-3.4 ± 0.58	-3.34 ± 0.26	0.4 (0.12,0.71)	Low	-8.85	0.22	0.30	0.14	Marginal
P2_Decmax	-6.03 ± 1.14	-5.8 ± 0.64	0.34 (0.07,0.66)	Low	-10.50	0.42	0.62	0.26	Marginal
P2_Decmax_Time	0.91 ± 0.2	0.86 ± 0.15	0.2 (0.03,0.54)	Low	16.09	0.09	0.14	0.05	Marginal
P2_Decmax_Dist	1.48 ± 0.31	1.38 ± 0.22	0.22 (-0.01,0.56)	Low	15.28	0.13	0.21	0.07	Marginal
P2_HBFavg	199.4 ± 43.7	195.81 ± 18.91	0.59 (0.33,0.83)	Moderate	9.92	16.19	19.93	12.69	Marginal
P2_HBFmax	356.79 ± 77.75	343.67 ± 41.27	0.52 (0.25,0.78)	Moderate	10.75	29.16	37.46	21.05	Marginal
P2_HBIavg	260.67 ± 39.26	258.61 ± 33.16	0.8 (0.61,0.92)	High	6.55	15.41	17.09	17.05	Marginal
P2_HBPavg	792.8 ± 163.45	773.06 ± 75.93	0.65 (0.4,0.86)	Moderate	8.58	57.46	68.27	48.68	Marginal
P2_HBPmax	1427.62 ± 346.71	1382.69 ± 216.81	0.55 (0.28,0.8)	Moderate	11.57	127.46	161.84	94.77	Marginal
P3_Time	1.4 ± 0.22	1.33 ± 0.15	0.38 (0.11,0.69)	Low	11.67	0.12	0.16	0.07	Marginal
P3_Dist	1.47 ± 0.33	1.38 ± 0.24	0.49 (0.22,0.77)	Low	15.97	0.18	0.23	0.13	Marginal
P3_Vavg	3.41 ± 0.28	3.36 ± 0.32	0.62 (0.36,0.84)	Moderate	5.54	0.15	0.19	0.13	Marginal
P3_Vmax	4.72 ± 0.34	4.61 ± 0.32	0.59 (0.32,0.82)	Moderate	5.26	0.21	0.24	0.16	Marginal
P3_Accavg	2.12 ± 0.42	2.15 ± 0.22	0.45 (0.17,0.74)	Low	11.09	0.18	0.23	0.12	Marginal
P3_Accmax	3.36 ± 0.57	3.57 ± 0.43	0.38 (0.1,0.7)	Low	12.32	0.34	0.43	0.22	Marginal
P3_HAFavg	129.46 ± 29.07	131.55 ± 18.51	0.62 (0.36,0.84)	Moderate	11.42	12.98	14.63	10.50	Marginal
P3_HAFmax	203.24 ± 39.86	215.51 ± 30.31	0.59 (0.32,0.82)	Moderate	11.84	22.12	24.92	17.23	Marginal
P3_HAIavg	178.92 ± 37.55	174.04 ± 22.2	0.64 (0.39,0.85)	Moderate	10.90	17.58	19.12	14.70	Marginal
P3_HAPavg	418.55 ± 80.24	420.52 ± 61.48	0.58 (0.32,0.82)	Moderate	11.18	40.79	45.72	31.62	Marginal
P3_HAPmax	676.2 ± 128.66	705.57 ± 82.62	0.44 (0.17,0.73)	Low	11.39	61.40	76.09	41.11	Marginal

**Table 4:** Inter-day reliability and sensitivity of phase-specific kinematic and kinetic metrics during the 505 change-of-direction test.

Variable	Day 1	Day 2	ICC (95% CI)	ICC magnitude	CV%	SEM	TE	SWC	Usefulness
P1_Time	2.42 ± 0.17	2.42 ± 0.17	0.67 (0.07,0.9)	Moderate	5.07	0.08	0.12	0.07	Marginal
P1_Dist	3.08 ± 0.19	3.01 ± 0.2	0.58 (0.24,0.86)	Moderate	4.83	0.10	0.15	0.08	Marginal
P1_Vavg	4.16 ± 0.2	4.07 ± 0.13	0.66 (0,0.89)	Moderate	2.74	0.09	0.11	0.07	Marginal
P1_Vmax	6.24 ± 0.15	6.24 ± 0.17	0.76 (0.23,0.92)	High	1.59	0.07	0.10	0.07	Marginal
P1_max_Time	2.42 ± 0.17	2.42 ± 0.17	0.67 (0.07,0.9)	Moderate	5.07	0.08	0.12	0.07	Marginal
P1_Accavg	2.59 ± 0.2	2.59 ± 0.23	0.76 (0.21,0.92)	High	5.30	0.09	0.14	0.10	Marginal
P1_Accmax	5.69 ± 1.2	5.38 ± 0.76	0.76 (0.28,0.92)	High	11.20	0.45	0.62	0.45	Marginal
P2_Time	1.32 ± 0.15	1.45 ± 0.13	0.58 (0.35,0.87)	Moderate	6.45	0.08	0.09	0.06	Marginal
P2_Dist	1.75 ± 0.21	1.86 ± 0.18	0.61 (0.12,0.87)	Moderate	7.45	0.10	0.13	0.08	Marginal
P2_Decavg	-3.36 ± 0.32	-3.06 ± 0.33	0.59 (0.34,0.88)	Moderate	6.42	0.19	0.21	0.14	Marginal
P2_Decmax	-5.89 ± 0.73	-5.17 ± 0.64	0.56 (0.37,0.87)	Moderate	7.90	0.41	0.44	0.31	Marginal
P2_Decmax_Time	0.88 ± 0.15	0.84 ± 0.13	0.17 (0,0.74)	Low	15.75	0.10	0.14	0.05	Marginal
P2_Decmax_Dist	1.42 ± 0.23	1.38 ± 0.19	0.36 (1.09,0.8)	Low	13.37	0.13	0.19	0.08	Marginal
P2_HBFavg	196.99 ± 26.37	178.1 ± 26.81	0.77 (0.13,0.94)	High	6.71	11.98	12.58	12.53	Marginal
P2_HBFmax	347.91 ± 52.25	304.78 ± 49.68	0.71 (0.27,0.92)	Moderate	7.97	25.54	26.02	23.77	Marginal
P2_HBIavg	257.48 ± 35.88	254.14 ± 32.42	0.9 (0.7,0.97)	Nearly Perfect	5.77	10.22	14.76	16.28	Good
P2_HBPavg	783.05 ± 105.3	710.96 ± 107.12	0.79 (0.09,0.95)	High	6.38	45.85	47.67	50.36	Good
P2_HBPmax	1397.56 ± 250.96	1305.38 ± 217.18	0.82 (0.41,0.94)	High	9.02	93.47	121.94	109.13	Marginal
P3_Time	1.37 ± 0.15	1.34 ± 0.14	0.74 (0.21,0.92)	Moderate	7.13	0.07	0.10	0.07	Marginal
P3_Dist	1.43 ± 0.24	1.39 ± 0.2	0.88 (0.64,0.96)	High	7.08	0.07	0.10	0.10	Good
P3_Vavg	3.39 ± 0.24	3.39 ± 0.26	0.87 (0.57,0.96)	High	3.64	0.08	0.12	0.12	Marginal
P3_Vmax	4.66 ± 0.29	4.61 ± 0.3	0.9 (0.69,0.97)	Nearly Perfect	2.78	0.09	0.13	0.14	Good
P3_Accavg	2.11 ± 0.33	2.07 ± 0.21	0.81 (0.42,0.94)	High	7.55	0.11	0.16	0.13	Marginal
P3_Accmax	3.43 ± 0.42	3.42 ± 0.52	0.82 (0.43,0.94)	High	7.82	0.18	0.27	0.22	Marginal
P3_HAFavg	128.68 ± 23.95	126.6 ± 18.81	0.89 (0.65,0.96)	High	7.72	6.81	9.85	10.19	Good
P3_HAFmax	206.91 ± 32.64	206.45 ± 38.51	0.91 (0.71,0.97)	Nearly Perfect	7.24	10.24	14.97	17.04	Good
P3_HAIavg	174.26 ± 30.81	168.15 ± 26.87	0.82 (0.47,0.94)	High	9.26	11.22	15.85	13.32	Marginal
P3_HAPavg	414.64 ± 68.69	410.23 ± 71.77	0.88 (0.62,0.96)	High	8.16	23.12	33.64	33.05	Marginal
P3_HAPmax	685.77 ± 101.15	658.95 ± 125.93	0.89 (0.68,0.97)	High	7.23	35.61	48.64	54.45	Good
P3_HAPmax	685.77 ± 101.15	658.95 ± 125.93	0.89 (0.68,0.97)	High	7.23	35.61	48.64	54.45	Good

### Intra-day reliability and sensitivity

Regarding kinematic variables, maximum velocity (P1_V max) demonstrated moderate intra-day reliability (ICC = 0.67, CV = 1.51%). Other metrics, such as Dec_Phase_Dist (ICC = 0.25, CV = 11.28%) and Dec_Phase_Decmax (ICC = 0.34, CV = 10.50%), exhibited low reliability (**Table 3**). Conversely, re-acceleration metrics such as ReAcc_Phase_Time (ICC = 0.48, CV = 14.47%) and ReAcc_Phase_Vavg (ICC = 0.50, CV = 6.97%) demonstrated only low to moderate reliability with increased variability. Among kinetic variables, P2_HBFavg showed moderate reliability (ICC = 0.59, CV = 9.92%), and P3_HAPavg also achieved moderate reliability (ICC = 0.58, CV = 11.18%), while P2_HBIavg displays high reliability (ICC = 0.8, CV = 6.55%) (**Table 3**). Crucially, while several variables demonstrated moderate to high relative reliability (ICC), their absolute usefulness was rated as ‘Marginal’. This indicates that the typical measurement error (TE) consistently exceeded the Smallest Worthwhile Change (SWC), making it challenging to confidently detect small, true intra-day performance variations.

### Inter-day reliability and sensitivity

In the inter-day assessment, P1_Vmax remained a robust kinematic indicator (ICC = 0.76, CV = 1.59%). Re-acceleration metrics, specifically P3_Time (ICC = 0.74, CV = 7.13%) and P3_Dist (ICC = 0.88, CV = 7.08%), also demonstrated high to very high reliability. In contrast, P2_Decmax_Time (ICC = 0.17, CV = 15.75%) and P2_Decmax_Dist (ICC = 0.36, CV = 13.37%) exhibited the lowest stability among metrics (**Table 4**). Exceptional reliability was observed for kinetic variables, particularly P2_HBIavg (ICC = 0.90, CV = 5.77%), P3_HAFmax (ICC = 0.91, CV = 7.24%), and P3_Vmax (ICC = 0.9, CV = 2.78%), all reaching ‘Nearly Perfect’ status (**Table 4**). To assess the usefulness of these metrics for detecting performance changes, typical error (TE) was compared against the smallest worthwhile change (SWC). Metrics were classified as ‘Good’ when TE≤SWC and ‘Marginal’ when TE>SWC. Several kinetic variables achieved ‘Good’ usefulness, specifically P2_HBIavg (TE 14.76<SWC 16.28), P2_HBPavg (TE 47.67<SWC 50.36), and P3_Vmax (TE 0.13<SWC 0.14). While other parameters exhibited high relative reliability (ICC), they were categorized as ‘Marginal’ because their measurement noise (TE) exceeded the SWC, potentially obscuring meaningful performance adaptations (**Table 4**).

## Discussion

The present study evaluated the intra-day and inter-day reliability of radar-derived mechanical properties during 505 tests in youth soccer players. Our results demonstrate that kinetic parameters—specifically horizontal braking impulse (HBIavg) and horizontal acceleration force (HAFavg)—yield high inter-day stability (ICC ≥ 0.89). Conversely, kinematic variables exhibited higher intra-day variability, particularly during the initiation and re-acceleration phases. This suggests that while the single-radar device (SRD) is robust for longitudinal monitoring of mechanical output, its precision in capturing discrete kinematic events within a single session remains sensitive to trial-to-trial movement inconsistencies.

The reliability observed for P1_Vmax (ICC 0.67–0.76) confirms that during the initial linear acceleration, pelvic displacement serves as a viable proxy for total body center-of-mass (COM) velocity (Samozino et al., 2016). This stability likely stems from the SRD’s single-point tracking of the pelvic region via established general mechanics models (Morin et al., 2012; Haugen and Buchheit, 2016; Samozino et al., 2016). Furthermore, this tracking consistency extends into the early deceleration phase; as braking represents the negative manifestation of acceleration and shares a linear movement pattern, radar-derived kinetic parameters showed good-to-excellent reliability (CV = 1.51–1.59%), mirroring our P1_Vmax results (Harper et al., 2023). These findings align with prior research utilizing SRD technology to quantify deceleration, which reported high reliability for metrics such as initial braking velocity (Harper et al., 2023; Chen et al., 2025). These observations reinforce the ecological validity of radar technology when assessing linear components of athletic performance.

However, the reliability of performance metrics tends to deteriorate as the task transitions from linear movement to the high-intensity 180°change-of-direction (Nimphius et al., 2018; Dugdale et al., 2020; Sánchez-López et al., 2023). Present results align with previous evidence indicating that deceleration and re-acceleration are inherently less reliable due to the absence of strict constraints on turn technique variations (e.g., foot placement angles) and asymmetrical limb loading during the plant phase (Jm and Wb, 2006; Nimphius et al., 2018; Dos’Santos et al., 2020). Firstly, the consideration of movement technique, such as turning style and foot placement, was not controlled, which may increase the variability in the mechanical calculation of deceleration parameters. Secondly, the players were asked to perform COD trials on the right leg, not the dominant leg, which may increase inter-individual movement variability due to a significant difference in 505 performance between the Dominant and Non-dominant legs (Clemente et al., 2022). As a result, this could increase the variability, especially the intra-day results, as mentioned earlier. Furthermore, the discrepancy between radar and 3D motion capture underscores the challenges of evaluating complex limb fluctuations using single-point tracking. In the m505 test, the 180-degree turn involves aggressive trunk inclination and rapid lowering of the COM. Because the SRD localized to the fixed height of the pelvic region cannot account for significant torso tilting or COM oscillations, a ‘biomechanical decoupling’ occurs between the pelvic sensor and the true whole-body kinematics. This explains why tracking stability was maintained during the initial 10-m acceleration but faltered during the high-velocity-to-zero transition, where the radar failed to capture instantaneous velocity shifts during the pivot.

The application of linear aerodynamic models to a 180-degree CoD task presents inherent mechanical challenges; the sensitivity of velocity capture during the turn renders the radar unable to resolve instantaneous changes when the COM shifts drastically (Jm and Wb, 2006; Nimphius et al., 2018). This limitation compromises the calculation of kinetic variables, such as Dec_Phase_HBFavg (ICC = 0.40). Similarly, the previous research validated MRD-derived velocity during the 505 test, noting that the biomechanical complexity of dynamic turns often leads to inconsistent reliability in kinetic and re-acceleration metrics (Eriksrud et al., 2022). Unlike systems employing a linear position transducer fixed to the waist—which may better approximate whole-body COM displacement—the single-point pelvic radar tracking appears more susceptible to error during dynamic maneuvers. Consequently, compared to trunk-mounted measurement systems, the reliability and validity of radar-derived parameters during the turning phase appear comparatively attenuated.

The observed variability across CoD phases is likely exacerbated by the SRD’s hardware specifications, specifically its sampling frequency. SRD technology typically operates at ~47 Hz, which is significantly lower than the >100 Hz resolutions of gold-standard motion capture and MRD assessment (1000Hz) (Eriksrud et al., 2022). Because the re-acceleration phase involves the redevelopment of explosive force within milliseconds, this lower sampling rate may fail to resolve transient peak kinetics. This technical bottleneck likely contributes to the elevated measurement error observed in P3_Vavg (CV = 5.54%) and P3_Time (Intra-day CV = 11.67%), while integrated-average metrics (e.g., P3_HBIavg) are inherently more trustworthy (ICC = 0.64-0.82, CV = 9.6-10.9). For youth athletes, the 180° turn poses a profound challenge to motor coordination rather than a purely physical capacity test, reflecting unique neuromuscular stressors (Rumpf et al., 2011; Dugdale et al., 2020). The current cohort may introduce biological “noise” due to ongoing neuromuscular development and fluctuating pacing strategies (Dugdale et al., 2020). Furthermore, while radar offers portability for field-based testing, the confluence of technical sampling limits and the inherent movement variability of youth athletes may inflate measurement error beyond the thresholds required for individualized monitoring (Rumpf et al., 2011). This necessitates a conservative, group-level interpretation of performance trends rather than isolated individualized diagnostics. The marked improvement in inter-day stability (e.g., P3_HAFavg improving from ICC 0.62 to 0.89) further suggests a familiarization effect, indicating that youth athletes require initial exposure to stabilize their motor patterns during the cognitively demanding 180° turn.

When interpreting radar-derived data, it is critical to distinguish between a metric’s ability to rank athletes (ICC) and its capacity to monitor true individual changes. While several variables showed high ICCs, their intra-day usefulness was universally “marginal” due to inherent trial-to-trial noise during the complex movement task, which we primarily attribute to a familiarization effect (Dos’Santos et al., 2018; Chen et al., 2025). In complex multi-planar movements like the 505-test, youth athletes often exhibit high technical variability during initial exposures. The lack of a prior dedicated practice session likely caused athletes to refine their braking and re-acceleration strategies throughout the first day’s trials. As a result, the intra-day assessments may have captured the initial stage of motor learning for the COD task. Conversely, achieving “Good” inter-day usefulness for key kinetic markers (e.g., P1_Vmax, P2_HBIavg, P2_HBFavg) suggests that movement patterns stabilized by the second session, making inter-day comparisons more reflective of true change in direction technique and resulting in higher reliability. For instance, in inter-day sessions, P2_HBIavg demonstrated ‘Good’ usefulness, with a typical error (14.76) lower than the SWC (16.28). As a result, despite kinematic limitations, the SRD remains a time-efficient field tool, provided practitioners prioritize metrics that are less sensitive to sampling fluctuations. However, for P1_Vmax, the inter-day TE (0.10) exceeded the SWC (0.07), implying that inherent noise may still mask small but meaningful individual improvements in elite youth cohorts. In a practical context, improvements in CoD technique may remain statistically indistinguishable from measurement error unless multiple trials are averaged to enhance the signal-to-noise ratio (Hewit et al., 2013; Dos’Santos et al., 2018; Nimphius et al., 2018). Practitioners should prioritize standardizing movement patterns and, where possible, complement radar data with additional biomechanical measures to mitigate the inherent variability typical of youth populations.

## Limitations

Several limitations warrant acknowledgment. A primary limitation of this study is the lack of a multi-day familiarization protocol. Second, the sample size may have lacked sufficient statistical power to detect nuanced effects. Thirdly, the exclusive focus on young male football players restricts generalizability to female athletes, broader age groups, and diverse sporting backgrounds. Finally, the absence of 3D motion capture and a force plate—the “gold standard”—precludes concurrent validation of radar-derived kinematic and kinetic metrics, potentially affecting the assessment of absolute accuracy. To address these gaps, future research should evaluate the reliability of radar-derived COD mechanics, including at least three dedicated practice sessions 24–48 hours prior to formal data collection to minimize learning-induced noise in intra-day assessments. And employ large-scale, multi-sport and level cohorts to enhance statistical power and establish reliability across diverse maturity and skill levels. Additionally, investigating the influence of fatigue, limb dominance, sex, and sport-specific reactive drills is essential to ensure the ecological validity of these metrics and examine technical constraints to minimize measurement noise.

## Conclusions

The current study suggested that Radar technology is a practical field-based tool for profiling COD mechanics in only male youth soccer, though its utility is primarily confined to inter-day monitoring. Despite high relative reliability (ICC > 0.70), intra-day usefulness remains “marginal” because trial-to-trial noise masks acute performance changes. Conversely, inter-day assessments of average braking impulse and peak re-acceleration velocity exhibit “Good” sensitivity (TE≤SWC), representing the most robust metrics for longitudinal tracking.

To effectively monitor change-of-direction mechanical parameters using a radar device, practitioners should prioritize inter-day variables—specifically P2_HBI avg, P3_V max, and P3_HAFavg. Since single-session noise can obscure genuine performance trends, it is essential to conduct at least 2 familiarization sessions or baseline tests (3–5 submaximal and maximal trials) 24–48 hours before the formal evaluation. This protocol ensures that subsequent data reflect the athlete’s true mechanical capacity rather than initial motor learning or technical stabilization. Decisions regarding training adaptations or readiness should be based on these stabilized inter-day trends to ensure measurement precision.

## Data Availability

The raw data supporting the conclusions of this article will be made available by the authors, without undue reservation.
